# Salmon Protein Hydrolysate Potentiates the Growth Inhibitory Effect of Bicalutamide on Human Prostate Cancer Cell Lines LNCaP and PC3 by Modulating Iron Homeostasis

**DOI:** 10.3390/md20040228

**Published:** 2022-03-28

**Authors:** Christian Bjerknes, Bomi Framroze, Crawford Currie, Caroline Hild Hakvåg Pettersen, Karol Axcrona, Erland Hermansen

**Affiliations:** 1 Hofseth Biocare, Kipervikgata 13, 6003 Ålesund, Norway; cc@hofsethbiocare.no; 2 GPH Biotech LLC, 1455 Adams Drive, Menlo Park, CA 94025, USA; 3 Department of Clinical and Molecular Medicine, Faculty of Medicine and Health Sciences, Norwegian University of Science and Technology, 7491 Trondheim, Norway; caroline.h.pettersen@ntnu.no; 4 Department of Urology, Akershus University Hospital, 1478 Lørenskog, Norway; axcrona@online.no; 5 Department of Clinical Medicine, University of Bergen, 5007 Bergen, Norway

**Keywords:** marine bioactive peptides, protein hydrolysate, prostate cancer, iron homeostasis

## Abstract

Prostate cancer is a common cause of cancer death in men. In advanced stages of prostate cancer, androgen deprivation therapy (ADT) is initiated. Despite ADT, prostate cancers invariably progress to become androgen independent. A growing body of evidence implicates iron dysmetabolism in prostate cancer progression. A bioactive peptide-rich salmon protein hydrolysate (SPH) has previously been demonstrated to modulate iron homeostatic mechanisms. In the present study, the anticancer effect of SPH and bicalutamide co-treatment on LNCaP and PC3 prostate cancer cell proliferation was investigated. Our results found that SPH potentiates the anti-proliferative effect of bicalutamide in a dose-dependent manner for both cell lines. In the presence of 160 µg/mL SPH, co-treatment with 1.0 µM bicalutamide decreased LNCaP cells’ relative colony survival from 25% (1.0 µM bicalutamide monotreatment) to 2% after culturing for 12 days. For PC3 cells, the relative colony survival diminished from 52% (10.0 µM bicalutamide) to 32% at an SPH concentration of 160 µg/mL. Gene expression profiling, employing quantitative real-time PCR, revealed that the inhibitory effects were related to significant FTH1 up-regulation with a concomitant TFRC down-regulation. In conclusion, our results provide in vitro evidence that SPH potentiates the growth inhibitory effect of bicalutamide on prostate cancer cells by modulating iron homeostasis mechanisms.

## 1. Introduction

Prostate cancer is the most prevalent cancer in men and was the fifth leading cause of cancer death in 2020 [1]. Despite being a common disease, its etiology remains obscure. Risk factors associated with prostate cancer include age, race, family history, and environmental influences. In recent years, the management of prostate cancer has rapidly evolved. A notable development is the estimated increase in prostate cancer incidence worldwide up to 2040 [2], likely related to increased life expectancy, increased reporting of cases, and a ‘westernized’ lifestyle characterized by physical inactivity and dietary factors [3]. Primary treatment for localized prostate cancer includes radical prostatectomy and radiation therapy. Metastatic prostate cancer is treated Androgen deprivation therapy (ADT), Androgen receptor inhibitors (ARIs), chemotherapy and radiation therapy. 

The dependence of prostate cancer progression upon androgens was recognized by Huggins and Hodges [4]. Since then, ADT for metastatic prostate cancer remains a cornerstone in the treatment of patients with advanced disease [5]. However, prostate cancer invariably develops resistance to ADT, resulting in castration-resistant prostate cancer [6]. Androgen resistance is partly caused by amplification and mutations of the androgen receptor that increase the activity of the androgen receptor (AR) pathway [7]. In the process of developing drugs for the treatment of prostate cancer, research on targeting of the AR has led to the discovery of ARIs. Bicalutamide [8], and more modern ARIs such as enzalutamide, darolutamide, and others, have been implemented in clinical use [9].

In the last decade, advances in our understanding of the pathophysiology of prostate cancer have implicated the dysregulation of iron homeostasis as an important driver of tumorigenesis [10]. Malignant cells typically rely heavily on iron for proliferation and spread, making them potentially more susceptible to iron depletion than normal cells [11]. Malignancies alter iron metabolism through processes of transferrin uptake, ferroportin export, altered hepcidin activity, and by modulating the cellular labile iron pool [12]. Frequently, malignant cells express high levels of transferrin receptors (TfR) which facilitate iron uptake [13]. Accordingly, TfR represents a possible target for cancer therapy [14].

Recent iron studies showed that high-dose iron induces ferroptosis in prostate cancer cell lines [15]. Subjecting malignant cells to high-dose iron disrupted the cells’ ability to adapt to an androgen deficient environment. High-dose iron was demonstrated to sensitize prostate cancer cells to the therapeutic action of Bicalutamide in vitro, implicating iron-regulatory mechanisms. Altered iron metabolism is also evident in other cancer types. Previous studies have linked down-regulated ferritin heavy chain 1 (FTH1) expression to cancer progression in breast- and colorectal cancer, suggesting a role for the ferritin subunit in tumor suppression [16,17]. A decreased FTH1 expression mediated by oncogenic microRNA (miRNA) has been reported in prostate cancer tissue samples. Moreover, levels of FTH1 expression have been demonstrated to relate to patient survival [18].

The ferritin genes belong to a family of genes which include pseudogenes. Pseudogenes have long been considered ‘vestigial DNA’, otherwise devoid of function. However, numerous studies have demonstrated evidence that FTH1 pseudogenes can be considered a regulator of important biological processes through their ability to bind and sequester miRNAs. Notably, FTH1 and its pseudogenes interact with the principal tumor suppressor p53, as demonstrated by FTH1 knockdown in mice that resulted in diminished p53 expression and translation. Moreover, in the context of prostate cancer and iron dysmetabolism, an FTH1 gene:pseudogene network appears capable of sequestering oncogenic miRNAs, interfering with competitively endogenous RNA (ceRNA) crosstalk, and effectively bearing out a tumor-suppressive function [18].

LNCaP and PC3 cells are two of the most commonly employed cell lines in prostate cancer research. LNCaP cells represent prostate cancer in its most indolent form. The cells still retain the ability to express AR and prostate-specific antigen (PSA) markers [19]. LNCaP cells are androgen-sensitive in that their further pathogenesis relies largely on stimulation by androgens. PC3 cells are another cell line characteristic of prostatic small cell carcinoma, a very aggressive and intractable variant of prostate cancer that typically does not respond to ADT. PC3 tumor cells are negative for AR and PSA.

Bioactive peptides (BAPs)—functional fragments of a parent protein that may regulate many important biological functions—have emerged as promising tools for future complementary medicine. BAPs have innate properties such as excellent tissue penetrability, broad specificity of action, and generally a good safety profile [20]. Research has shown that specific activator peptides present in a salmon protein hydrolysate (SPH) increase the expression of FTH1 gene within human gingival epithelial cells [21]. In a clinical trial, iron-deficient anemic individuals treated with the same salmon protein hydrolysate for 6 weeks showed a 14% increase in hemoglobin concentration [22]. Considering the importance of iron dysmetabolism in prostate cancer, we applied these findings to examine the effects of co-treatment with SPH and bicalutamide on the proliferation of LNCaP cells and PC3 cells. A differential gene expression analysis with respect to FTH1 and TFRC gene expression was performed. 

## 2. Results

### 2.1. Effects of SPH Monotreatment on the Proliferation of LNCaP and PC3 Cells

**Figure 1:**
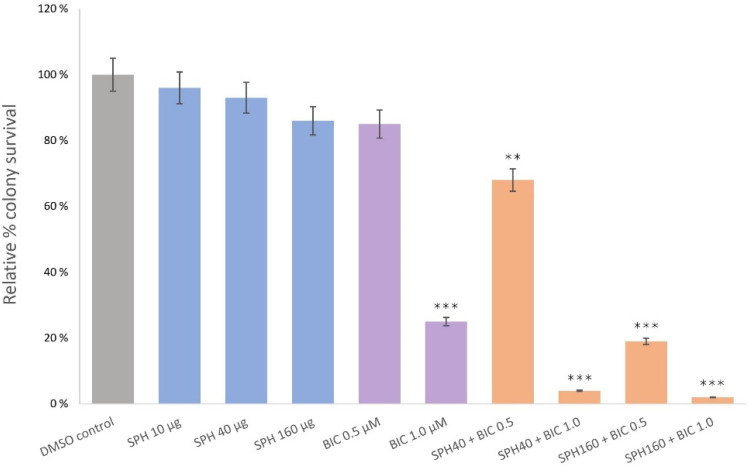
Clonogenic assay illustrating LNCaP relative colony survival with varying doses of SPH, bicalutamide (BIC), or co-treatment with SPH and BIC. Treatments were applied daily for 5 days without changing the media. Post-12-day culturing cells were quantified with an automatic cell counter. *p* values less than 0.01 are summarized with two asterisks, and *p* values less than 0.001 are summarized with three asterisks.

LNCaP and PC3 cells were exposed for five days to SPH test solutions containing 10 µg, 40 µg, and 160 µg SPH. Colonies were counted using a TC20 Automated Cell Counter. Although a numerical reduction in formed LNCaP colonies was observed after incubation with SPH, relative to the solvent control (0.1% DMSO), inhibition of cell proliferation did not reach statistical significance at all tested concentrations of SPH in this specific cancer cell line (Figure 1). Similarly, for PC3 cells, no statistically significant change in cell survival was observed at any of the tested concentrations. 

### 2.2. SPH and Bicalutamide Co-Treatment Significantly Reduces Relative Colony Survival of AR-Positive LNCaP Prostate Cancer Cells

We investigated whether bicalutamide in combination with SPH would mitigate the rate of colony formation in AR-positive LNCaP prostate cancer cells in a synergistic manner. The tumor suppressive capacity of bicalutamide is well established, and in this study was statistically significant at concentrations of 1 µM (*p* < 0.001), although not at 0.5 µM. Co-treatment with SPH lowered the minimum inhibitory concentration (MIC) for bicalutamide at SPH doses of both 40 µg/mL and 160 µg/mL, demonstrating a stronger anti-proliferative effect at a given concentration of bicalutamide when co-treated with SPH (Figure 1). 

Relative colony survival decreased from 85% (bicalutamide monotreatment at 0.5 µM) to 68% when 40 µg/mL SPH was co-administered with 0.5 µM bicalutamide. Furthermore, LNCaP cell viability significantly decreased to 19% with an SPH concentration of 160 µg/mL (*p* < 0.001). Relative colony survival decreased from 25% (bicalutamide monotreatment at 1.0 µM) to 4% when 40 µg/mL SPH was co-administered with 1.0 µM bicalutamide. The highest anti-proliferative effect was observed after the incubation of LNCaP cells in the presence of 160 µg/mL SPH with 1 µM bicalutamide, where the relative colony survival decreased from 25% (as seen with 1.0 µM bicalutamide monotreatment), to 2% (*p* < 0.001). 

### 2.3. SPH and Bicalutamide Co-Treatment Significantly Reduces Relative Colony Survival of AR-Negative PC3 Prostate Cancer Cells

**Figure 2:**
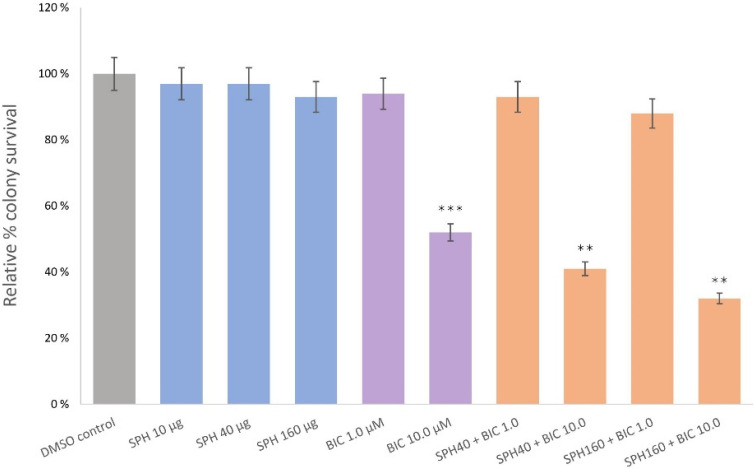
Clonogenic assay illustrating PC3 relative colony survival with varying doses of SPH, bicalutamide (BIC), or co-treatment with SPH and BIC. Treatments were applied daily for 5 days without changing the media. Post-12-day culturing cells were quantified with an automatic cell counter. *p* values less than 0.01 are summarized with two asterisks, and *p* values less than 0.001 are summarized with three asterisks.

We next investigated the effect of SPH and bicalutamide co-treatment on PC3 prostate cancer cells. Bicalutamide monotreatment at 1.0 µM was ineffective in reducing cancer cell proliferation. PC3 cells were partially sensitive only at higher bicalutamide concentrations (10 µM; Figure 2), which resulted in a reduction in relative colony survival to 52% (*p* < 0.001). At the non-effective bicalutamide concentration of 1.0 µM, co-treatment with SPH at 40 and 160 µg/mL, respectively, demonstrated no significant reduction in the colony formation rates of PC3 cells. However, the experiment found that incubation with 10 µM bicalutamide and 40 µg/mL SPH significantly diminished relative colony survival from 52% to 41% (*p* < 0.001), with an additional decrease to 32% when the SPH concentration was increased from 40 to 160 µg/mL (*p* < 0.01). The cytostatic effect of co-treatment was overall weaker on PC3 cells, than that exerted on LNCaP cells. 

### 2.4. Gene Expression Profiles of AR-Positive LNCaP and AR-Negative Prostate Cancer Cells

Differential gene expression profiles with respect to iron homeostasis were evaluated post-treatment with bicalutamide monotreatment, and bicalutamide and SPH co-treatment. A two-fold change in gene expression was necessary to reach statistical significance. Gene expression profiling revealed that the treatment of both LNCaP and PC3 cells with SPH and bicalutamide induced a significant FTH1 up-regulation by greater than two-fold, as well as a concomitant TFRC down-regulation by greater than two-fold (Table 1). SPH monotreatment of both LNCaP and PC3 cells led to numerical changes in gene expression; however, the changes were not statistically significant.

## 3. Discussion

In this article, we tested the effect of a salmon protein hydrolysate and/or bicalutamide at a range of set concentrations in prostate cancer cells in vitro. 

We have previously shown that certain peptides found in SPH are capable of up-regulating FTH1 and down-regulating TFRC in normal human gingival epithelial cells [21]. Given that iron dysmetabolism has been demonstrated to play an important role in driving the pathogenesis of prostate cancers [23], as well as recent findings implicating FTH1 as having nontrivial tumor suppressive properties [24], we sought to expose LNCaP AR-positive and PC3 AR-negative prostate cancer cells to SPH and/or bicalutamide to investigate whether, and to what extent, this strategy would inhibit cancer cell proliferation via gene regulation.

Iron is a fundamental microelement necessary for tumor growth. A normal cellular iron balance is maintained by the coordinated regulation of the storage protein ferritin and iron transporters, including the importer transferrin receptor-1 (TfR1) and the exporter protein ferroportin [25]. Prostate cancers favor iron retention, as it has been shown that prostate cancer cells regulate local hepcidin synthesis in an autocrine manner, resulting in ferroportin degradation, which facilitates the intracellular accumulation of free iron [23]. Moreover, increased levels of the TfR1 importer protein, encoded by the TFRC gene, have been reported in prostate cancer cell lines, improving intracellular iron uptake [23]. FTH1 and its pseudogenes are often suppressed in prostate cancer tissue samples [18], and this is suggested to occur through oncogenic miRNA-mediated down-regulation. 

FTH1 down-regulation is likely an adaptive response, as there is evidence to suggest that FTH1 expression is innately tumor suppressive. Recent observations in preclinical breast cancer models suggest that FTH1 is a significant tumor suppressor by inhibiting the expression of key oncogenes, such as c-MYC, and that its increased expression signifies a favorable prognosis and response to chemotherapy [24]. Increasing FTH1 expression would likely restore its function as an iron regulator and tumor suppressor by sequestering oncogenic miRNAs, relieving it of oncogenic miRNA inhibition. An increase in FTH1 sequesters free iron, which reduces the growth of prostate cancer cells. 

Our results demonstrate that SPH synergizes with bicalutamide, as bicalutamide was more effective at a given dose when co-administered with SPH. SPH as a monotherapy did not, however, exhibit statistically significant growth inhibiting effects on either cell line, even though numerical differences were observed. Differential gene expression analysis found that FTH1 was up-regulated and TFRC was down-regulated by greater than 2-fold versus the housekeeping ACTB gene expression. This pattern of gene expression would rearrange iron-trafficking and storage proteins in a way that is unfavorable to cancer survival, through an increase in FTH1-mediated tumor suppression and iron sequestration, while diminishing the ability of iron uptake through the TfR1 importer. In light of recent work implicating FTH1 in the prognosis of breast cancer [24], we can speculate whether similar outcomes could be observed in cancers such as breast cancer cells if exposed to SPH and an appropriate breast cancer therapeutic. 

As outlined before, LNCaP express the AR and are sensitive to androgen inhibitors, while PC3 cells frequently lack AR entirely, and are a preclinical model of aggressive prostate cancer. PC3 cells were only sensitive to bicalutamide at higher concentrations. Exposure to SPH did make PC3 cells receptive to the action of bicalutamide at lower concentrations, effectively sensitizing the cell to its therapeutic action. 

The issue of iron metabolism in cancer remains incompletely understood, but iron metabolism is proposed to be linked to active androgen signaling [15]. More research is needed to elucidate, in detail, the mechanistic underpinnings observed here, but certainly a differential FTH1 and TFRC gene expression is implicated in SPH’s mode of action in this study. It would be useful to repeat this experiment in similar prostate cancer cell lines such as AR-positive VCaP cells and androgen-insensitive DU-145 cells. Further studies are warranted, specifically changes in signaling proteins involved in cell proliferation and apoptosis in LNCaP and PC3 cell lines, as well as changes in cell cycle growth phases. Finally, it would be beneficial to investigate changes in the cells’ labile iron pool. Importantly, the results need to be validated in vivo employing LNCaP and PC3 xenografts by investigating SPH and/or bicalutamides’ effect on tumor volume. For future clinical trials employing bioactive peptides, consideration must be given to the principle obstacles of bioactive peptides, which typically include low oral bioavailability, low plasma stability, and short circulation time [26]. Bioactive peptides must be resistant to degrading peptidases present in the gastrointestinal tract, the intestinal brush border, and be able to circulate stably in serum in order to reach their target active site to exert a function. Because of their frequent molecular steric factors, marine-derived peptides can be innately more resistant to such degradation, which may present an advantage over other peptide sources [27]. 

We hope the current work can shed some light on what is a complex although important challenge to address, and aid in developing novel therapeutic strategies aimed at optimizing drug efficacy and overcoming androgen-resistance. 

## 4. Materials and Methods

### 4.1. Materials and Reagents

The human prostatic carcinoma cell lines for in vitro assays LNCaP and PC3 were obtained from the American Type Culture Collection(Manassas, VA, USA); RPMI cell culture medium 1640 was purchased from Lonza Bioscience (Morrisville, NC, USA); L-glutamine, penicillin, streptomycin and 10% heat-inactivated Fetal Bovine Serum (FBS) were purchased from Thermo Fischer Scientific (Waltham, MA, USA); and ≥98% Bicalutamide powder, Dimethyl sulfoxide (DMSO) and Dulbecco’s Modified Eagle’s Medium (DMEM) were from Sigma-Aldrich (St. Louis, MO, USA). Salmon protein hydrolysate (SPH) was obtained from Hofseth Biocare ASA, Ålesund, Norway. The SPH was manufactured by a proprietary enzymatic hydrolysis technology, with a degree of enzymatic hydrolysis of 10%. It appears as a light yellow powder, with a water soluble protein content of >95% (>25% type I/III collagen peptides), fat content <0.5% and ash content <2.5%. SPH contain both essential and conditionally essential amino acids. It contains particularly high amounts of glutamic acid (13.9 g/100 g), aspartic acid (9.4 g/100 g), glycine (14.9 g/100 g), proline (7.6 g/100 g), lysine (7.0 g/100 g), alanine (7.5 g/100 g) and arginine (6.9 g/100 g). Molecular weight distribution analysis show that 41.6% of the water soluble hydrolysate is composed of peptides less than 4000 D. Cells were tested negative for *Mycoplasma*.

### 4.2. Cell Culture Preparation

LNCaP and PC3 cells were maintained in RPMI 1640 supplemented with 2 mmol/L L-glutamine, 100 U/mL penicillin, 100 mg/mL streptomycin, and 10% heat-inactivated FBS. Cells were grown at 37 °C in a humidified 5% CO_2_-atmosphere. 

### 4.3. Test Solutions

SPH test solutions were prepared with 10 µg, 40 µg, and 160 µg of SPH powder in 1 mL of DMEM containing 0.3% FBS and 2% DMSO, sonicated for 10 min prior to use. Bicalutamide (Sigma-Aldrich, St. Louis, MO, USA; catalogue no. B9061) test solutions were made containing 0.43 µg and 4.3 µg in 1 mL of DMEM containing 0.3% FBS and 2% DMSO for a final concentration of 1 µM and 10 µM, and finally sonicated for 10 min prior to use. 

### 4.4. Colony Formation Assay

To test cell proliferation, confluent cells were trypsinized and seeded onto 10-mm Petri dishes at a density of 3000–5000 cells according to the manufacturer’s protocol with respect to the different cell lines. After 24 h, cells were treated with the indicated concentrations of either SPH alone, bicalutamide alone, or a co-treatment of SPH and bicalutamide. To determine the dose of bicalutamide, the MIC for bicalutamide was established using already published data [28,29]. Treatments were applied daily for 5 days without changing the media. Excess volume of test solution applied to the media was pipetted off. After culturing for 12 days, cells were stained with crystal violet and counted employing a TC20 Automated Cell Counter (Bio-Rad; Hercules, CA, USA). Using untreated cells (cell media + 0.1% DMSO), each assay was internally controlled. Relative plating efficiencies were expressed as percentages relative to the plating efficiency of untreated cells and reported as the relative percent colony survival rate. The experiments were performed in triplicate.

### 4.5. Gene Expression Assay

Samples were studied with respect to FTH1 and TFRC gene expression. Total RNA was isolated from cells with a one-step liquid phase separation using UPzol™ RNA Isolation Solution (Biotechrabbit, Berlin, Germany). DNA contamination was cleared using TURBO™ DNase, according to the manufacturer’s protocol. Quantitative conversion of total RNA to single-stranded cDNA was achieved employing the high-capacity cDNA Reverse Transcription Kit (Applied Biosystems; Waltham, MA, USA). Levels of gene expression were measured by qRT-PCR. Then, 1 µL of cDNA (corresponding to 50 ng of reverse transcribed RNA) was amplified using quantitative real-time PCR (QuantStudio™ 6 Flex Real-Time PCR System), using a TaqMan™ Universal PCR Master Mix (Catalog number: 4304437), and a TaqMan Assay (Thermo Fisher Scientific; Waltham, MA, USA).

Gene expression was estimated relative to housekeeping ACTB expression following the formula 2^−ΔCt^.

TaqMan probe Ids used were: (i) FTH1—Hs01694011_s1; (ii) TFRC—Hs00951083_m1; (iii) Housekeeping ACTB—Hs01060665_g1.

### 4.6. Statistical Analysis

Statistical difference between means between treatment groups in each cell line were determined using a paired, two-tailed Student’s *t*-test. 

## 5. Conclusions

In the present study, a bioactive peptide-rich SPH potentiated the antiproliferative activity of the competitive AR antagonist bicalutamide in two in vitro prostate cancer models. SPH co-administered with bicalutamide demonstrated the significant up-regulation of FTH1 and down-regulation of TFRC, a gene expression profile unfavorable for further prostate cancer cell growth. Further research is warranted to confirm and validate the effects observed here, and whether the findings are translatable and replicable in an in vivo model such as LNCaP and PC3 xenografts needs confirmation. Further work is needed to determine whether SPH has potential as an adjunct to a therapeutic regime for advanced-stage prostate cancer.

## Figures and Tables

**Table 1:** Gene expression assay results showing the effect of varying doses of monotreatment with bicalutamide (BIC) and co-treatment with BIC and SPH on the gene expression of FTH1 and TFRC genes in LNCaP and PC3 cells. Significant (>2-fold) up-regulation of FTH1 gene expression was observed in both LNCaP and PC3 cells. Significant (>2-fold) down-regulation of TFRC gene expression was observed in both LNCaP and PC3 cells.

Cell Line	Treatment	Gene	Fold Change			Average	SD
LNCaP	1 µM BIC	FTH1	1.0	1.1	1.0	1.0	0.1
	1 µM BIC	TFRC	1.0	1.0	1.2	1.1	0.1
LNCaP	40 µg/mL + 1 µM BIC	FTH1	2.3	2.2	2.3	2.3	0.1
	40 µg/mL + 1 µM BIC	TFRC	0.5	0.6	0.5	0.5	0.1
LNCaP	160 µg/mL + 1 µM BIC	FTH1	2.8	3.0	2.7	2.8	0.2
	160 µg/mL + 1 µM BIC	TFRC	0.3	0.4	0.5	0.4	0.1
PC3	10 µM BIC	FTH1	0.9	1.0	1.2	1.0	0.2
	10 µM BIC	TFRC	1.0	1.1	1.2	1.1	0.1
PC3	40 µg/mL + 10 µM BIC 40 µg/mL + 10 µM BIC	FTH1 TFRC	2.2	2.4	2.5	2.4	0.2
			0.5	0.5	0.4	0.5	0.1
PC3	160 µg/mL + 10 µM BIC 160 µg/mL + 10 µM BIC	FTH1 TFRC	2.8	2.6	2.4	2.6	0.2
			0.3	0.3	0.4	0.3	0.1

## Data Availability

All data is contained within the article.
